# Multi-level responses of *Camellia reticulata* to heat stress: insights from photosynthetic performance, antioxidant activity, lipidomics, and fatty acid profiling

**DOI:** 10.1186/s12870-026-09072-x

**Published:** 2026-06-15

**Authors:** Aiguo Jiang, Xiaoqian Sun, Xian Liu, Sijing Wang, Yanling Zheng

**Affiliations:** 1 Key Laboratory for Forest Resources Conservation and Utilization in the Southwest Mountains of China, Ministry of Education, Southwest Forestry University, Kunming, Yunnan 650224 China; 2 Yunnan Academy of Biodiversity, Kunming, Yunnan 650224 China; 3 Key Laboratory of Forest Disaster Warning and Control in Yunnan Province, Southwest Forestry University, Kunming, Yunnan 650224 China

**Keywords:** *Camellia reticulate*, Photochemical activity, Fatty acids, Lipidome, Thermotolerance

## Abstract

**Background:**

Climate change-induced heatwaves threaten the sustainable cultivation of *Camellia reticulata*, a valuable oil-producing species adapted to cool climates. Despite its economic importance, the mechanisms underlying its response to elevated temperatures are poorly understood. We hypothesized that *C. reticulata* activates distinct physiological and lipidomic adjustments to cope with different intensities of heat stress. To test this hypothesis, we subjected plants to three temperature regimes: 25 °C (control), 35 °C, and 40 °C, and conducted an integrated analysis of physiological traits and lipid metabolism.

**Result:**

Our results revealed distinct response strategies in *C. reticulata* under moderate versus extreme heat stress. Both 35 °C and 40 °C treatments led to significant decreases in chlorophyll content compared with the control, with a much more drastic reduction under 40 °C. Exposure to 35 °C enhanced antioxidant systems and shifted membrane lipids toward a more thermostable composition, effectively preventing visible injury despite mild PSII photoinactivation. In contrast, plants exposed to 40 °C displayed severe PSII photodamage and progressive leaf browning during recovery, even with elevated flavonoid accumulation and enhanced antioxidant capacity. Buds, however, remained viable. This physiological disruption was closely linked to deleterious remodeling of membrane lipids that compromises membrane stability. Concomitantly, significant accumulation of unsaturated neutral glycerolipids was observed, suggesting a cellular protective mechanism that sequesters excess unsaturated fatty acids into storage pools. The preferential survival of buds over leaves indicates that plants prioritize meristem protection. This study identifies the adaptive mechanisms under moderate heat and characterizes the physiological breakdown under extreme heat, highlighting lipid metabolism as a key target for improving thermotolerance in *C. reticulata*.

**Supplementary Information:**

The online version contains supplementary material available at 10.1186/s12870-026-09072-x.

## Introduction

The genus *Camellia* (Theaceae) is a key component of subtropical evergreen broad-leaved forests in China and holds immense economic importance as a source of woody oil, tea, and ornamental plants. However, this valuable genus is highly vulnerable to heat stress, with exposure to temperatures above 35 °C typically leading to leaf scorching, bud abortion, and flower drop [1]. The heat threat is potentially intensified by ongoing climate change [2]. In China, a clear trend of intensifying temperatures and more frequent extreme heatwaves has been observed [3], with some regions already experiencing consecutive days above 40 °C. Consequently, thermotolerance may become a critical constraint for both the conservation of these forest ecosystems and the sustainable cultivation of economically vital *Camellia* species. While interspecific variation in heat tolerance has been documented, the underlying metabolic mechanisms remain largely unexplored across the genus, with the notable exception of the model crop *C. sinensis* [4–7]. The lack of mechanistic understanding is particularly pronounced for *C. reticulata*, an important ornamental species and woody oil plant, the seeds of which contain high levels of unsaturated fatty acids [8]. Native to the cool, humid montane regions of southwestern China [9], *C. reticulata* faces a growing threat from heat stress. Despite being identified as relatively heat-tolerant [6], the specific metabolic basis of its resilience is completely unknown.

Plant responses to high temperature typically involve a cascade of interconnected physiological and biochemical events. A primary and highly sensitive target is the photosynthetic apparatus [10–12]. Elevated temperatures alter the conformation and activity of key photosynthetic complexes, disrupting the precise balance between light energy absorption and its utilization in carbon fixation [11, 13]. To mitigate the resulting energy overload, plants employ photoprotective mechanisms such as non-photochemical quenching, which safely dissipates excess energy as heat and protect the photosystems from damage [14]. The efficiency of these photochemical and photoprotective processes is most sensitively monitored in vivo through chlorophyll fluorescence kinetics, providing a real-time diagnostic for the status of the photosynthetic apparatus [12, 15].

However, when photoprotection is insufficient, the excess energy drives the over-reduction of electron carriers, forcing electrons onto alternative pathways that generate reactive oxygen species (ROS), thereby initiating a state of oxidative stress [10, 16]. The polyunsaturated fatty acyl chains of membrane lipids are a major target of oxidative damage, particularly in chloroplast envelopes and thylakoids, which are rich in these lipids [17, 18]. This oxidative attack triggers lipid peroxidation, the products of which can damage photosystem II (PSII) [19]. Among these products, malondialdehyde (MDA) commonly employed as a biomarker of oxidative damage to membrane systems [20, 21]. Photosynthetic pigments themselves are also susceptible to oxidative stress, leading to a decline in their content, which directly impairs light harvesting and photosynthetic capacity [13, 22]. Confronted with this oxidative challenge, plants deploy a multi-layered antioxidant defense, including both enzymatic components and a suite of non-enzymatic, redox-active metabolites [23]. Among the latter, flavonoids are notably induced under stress and serve a key function in re-establishing redox balance at the cellular level [24].

As the fundamental structural components, lipids directly determine key membrane biophysical properties, such as fluidity and permeability [25, 26]. Given that the photosynthetic apparatus is a primary target of heat, thylakoid membrane lipid composition plays a critical role in sustaining electron transport efficiency and the stability of complexes such as PSII [13, 27, 28]. Under heat stress, a common adaptive strategy involves the remodeling of these thylakoid membrane lipids, which includes reducing their unsaturation and decreasing the ratio of monogalactosyldiacylglycerol to digalactosyldiacylglycerol (MGDG/DGDG) [29, 30]. These compositional shifts collectively lower membrane fluidity and enhance bilayer stability, thereby counteracting heat-induced disorder and preventing leakiness [29, 30]. This targeted and multifaceted lipidomic reprogramming is a core acclimation strategy directly linked to enhanced thermotolerance [31, 32].

Therefore, an integrated analysis of photosynthetic physiology (including pigment content and chlorophyll fluorescence), oxidative stress markers, antioxidant capacity, and lipidome dynamics offers a comprehensive approach to elucidate the metabolic basis of plant thermotolerance. Given that most *Camellia* species exhibit physiological injury at temperatures exceeding 35 °C [1], we set 35 °C as an upper acclimation threshold to probe the limits of its adaptive capacity. Concurrently, to evaluate its resilience under future climate extremes, 40 °C was selected as a severe stress treatment. Based on this framework and our preliminary observations of *C. reticulata*’s recovery after extreme high-temperature stress, we hypothesize that its thermotolerance is conferred by a robustly capacity to manage ensuing oxidative stress and execute a stabilizing reorganization of its membrane lipid profiles. This study employs integrated physiological and lipidomic analyses to test this hypothesis and elucidate the metabolic foundation of thermotolerance in *C. reticulata*, providing insights vital for its conservation and climate-resilient cultivation.

## Materials and methods

### Experimental materials and treatments

The one-year-old *C. reticulata* seedlings were sourced from the Forestry and Grassland Technology Extension Station of Baoshan City, Yunnan Province, China. Prior to the experiment, all seedlings were acclimatized for six months in a greenhouse. They were cultivated in plastic pots (12 cm in diameter, 11 cm in height) containing a 1:1 (v/v) mixture of humus and laterite soil. During acclimation, the greenhouse conditions were maintained at a day/night temperature of 30/20 °C and a photosynthetic photon flux density of 600 μmol m^−2^ s^−1^. The seedlings were watered every 2–3 days.

The heat treatment experiment was conducted in programmable growth chambers (LISK, RGD-DW550C). The chambers were set to a 12-h light/12-h dark photoperiod, a light intensity of 600 μmol m^−2^ s^−1^, and a relative humidity of 60 ± 5%. A completely randomized design was applied with three temperature regimes: 25 °C (Control), 35 °C (H35), and 40 °C (H40), with each constant temperature treatment lasting for 3 days. This specific gradient and duration, chosen based on preliminary experiments, were sufficient to produce a gradation of physiological responses for comparison. Chamber temperatures were precisely maintained by digital control systems. To maintain plant hydration and minimize micro-environmental variation, all plants were watered daily to container capacity (until drainage commenced) and pots were randomly repositioned each day. Each treatment group comprised 20 plants.

Immediately following the treatment, sampling and measurements were performed. For chlorophyll fluorescence measurement, the third and fourth fully expanded leaves taken from every one of five randomly chosen plants in each treatment were measured. The average value for each plant was calculated and considered one biological replicate (*n* = 5). For destructive biochemical analyses, the same leaf positions (third and fourth) were collected from the other 15 plants in each treatment. To form each of the five composite biological replicates (*n* = 5), leaf tissue from three plants was pooled. Thus, 15 plants were used for biochemical and lipidomic analyses (3 plants × 5 replicates), which, together with the 5 plants used for chlorophyll fluorescence measurements, accounts for all 20 plants per treatment group. This pooling strategy was adopted for the destructive biochemical and lipidomic analyses to reduce within-treatment variability and obtain a representative metabolic profile for each treatment group. All collected leaf samples were immediately snap‑frozen in liquid nitrogen and then kept at − 80 °C until further analysis.

### Measurement of chlorophyll fluorescence parameters

Prior to measurement, all plants were subjected to a 30 min dark adaptation [33]. Chlorophyll fluorescence measurements were conducted on 2 leaves per plant using a Walz PAM-2500 modulated fluorometer (Germany), with ten biological replicates. The actinic light intensity was set at 350 μmol·m^−2^·s^−1^, and the plants were adapted to this light for more than 5 min. The saturating light pulse was set at approximately 8000 μmol·m^−2^·s^−1^ for 0.8 s. The maximum quantum efficiency of PSII (Fv/Fm), the effective quantum yield of PSII [Y(II)], and the non-regulated [Y(NO)] and regulated [Y(NPQ)] non-photochemical energy dissipation in PSII were analyzed. The parameters were calculated using the following equations [34]:Fv/Fm = (Fm − Fo)/Fm;Y(II) = (Fm′ − Fs)/Fm′;Y(NPQ) = Fs/Fm′ − Fs/Fm;Y(NO) = Fs/Fm. where Fo and Fm are the dark-adapted minimum and maximum fluorescence, respectively; Fs is the light-adapted steady-state fluorescence; and Fm′ is the light-adapted maximum fluorescence.

### Quantification of key biochemical parameters

Photosynthetic pigments, MDA, and Total antioxidant capacity (TAC): chlorophyll content was quantified followingwith five biological replicates [35]. TAC and MDA levels were measured using FRAP-2-G and MDA-2-Y kits (Comin Biotechnology, China) following manufacturer protocols, with five biological replicates per treatment.

Flavonoids: The frozen samples were allowed to thaw at 4 °C. Then, 100 mg of each specimen was combined with 10 μL of isotopically labeled internal standards (10 μg/mL) and 300 μL of ice‑cold methanol/aqueous solution (7:3, v/v). The resulting mixture was vigorously vortexed and subsequently sonicated in a water bath for 0.5 h. After centrifugation at 14,000 rcf (4 °C) for 20 min, the supernatant was filtered using an Ostro 25 mg 96-well plate with a positive pressure device. The filtrate was collected into a 2 mL EP tube and stored at − 80 °C until analysis. Flavonoids were quantified using high-performance liquid chromatography-tandem mass spectrometry (HPLC–MS/MS). Retention times and chromatographic peak areas were derived from MultiQuant software; external standard-based retention time calibration was subsequently used for metabolite identification.

### Analysis of lipid profile

The non-targeted lipid profile and targeted fatty acid profile were analyzed according to the method described in [36]. Specifically, freeze-dried samples were homogenized and subsequently mixed with 200 µL of ice-cold deionized water and 800 µL of chilled methyl tert-butyl ether (MTBE), followed by 30-s vortexing. The resulting mixture was then combined with 240 µL of methanol, subjected to 20-min sonication at 4 °C, allowed to stand for 30 min, and centrifuged at 14,000 × g for 15 min at 10 °C. The upper organic phase was collected and concentrated using a vacuum centrifuge. Prior to LC–MS analysis, lipid extracts were reconstituted in 200 µL of isopropanol:acetonitrile (9:1, v/v) solution. Chromatographic separation was achieved using a Waters ACQUITY PREMIER CSH C18 column (1.7 µm particle size, 2.1 mm internal diameter × 100 mm length). Solvent A was acetonitrile–water (6:4, vol/vol) containing 0.1% formic acid and 0.1 mM ammonium formate, and solvent B was acetonitrile–isopropanol (1:9, vol/vol) containing 0.1% formic acid and 0.1 mM ammonium formate. The gradient elution was programmed as follows: the initial mobile phase contained 30% solvent B and 70% solvent A at a flow rate of 300 μL/min. It was held for 2 min, and then solvent B was linearly increased to 100%, while solvent A was decreased to 0% from 2–25 min. This was followed by equilibration in 5% solvent B and 95% solvent A from 25–35 min. Mass spectrometry analysis was performed using a Q-Exactive Plus instrument (Thermo Fisher Scientific) operating in both positive and negative electrospray ionization (ESI) modes. The ESI source parameters were set as follows: heater temperature, 300 °C; capillary temperature, 350 °C; spray voltage, 3.0 kV; and S-Lens RF level, 50%. Full-scan MS1 spectra (range: m/z 200–1800) were acquired at a resolution of 70,000 (at m/z 200). Data-dependent acquisition (DDA) was employed, whereby the top 10 most intense ions from each full scan were selected for fragmentation via higher-energy collisional dissociation (HCD), with MS2 spectra collected at a resolution of 17,500 (at m/z 200).

Lipid identification was conducted using the LipidSearch software (Thermo Fisher Scientific, USA). Identifications were considered reliable based on the following criteria: (1) a precursor mass tolerance of 5 ppm, (2) an MS/MS spectral match with a product ion tolerance of 5 ppm and a product ion intensity threshold of 5%, and (3) a chromatographic retention time consistent with that of authentic standards or the expected elution order for each lipid class. Only identifications meeting all these criteria were accepted for further data analysis.

The double bond index (DBI) was calculated according to the formula published from [37], where N denotes the number of double bonds per lipid molecule. DBI = (∑[N × amount% lipid])/100.

### Determination of fatty acid profile

The total fatty acid (FA) profile was determined as described in detail [36]. In brief, the freeze-dried samples were ground and a solution of chloroform:methanol (3:2, v/v) was used to extract FAs. After ultrasonication for 30 min, 2 mL of 1% sulfuric acid in methanol were added to the supernatant. For methyl esterification, the mixture was incubated in an 80 °C water bath for 30 min to achieve FA esterification. Then, 1 mL n-hexane and 5 mL water were added and vortex-mixed. The supernatant (500 µL) was spiked with an internal standard (25 µL of 500 ppm methyl salicylate), mixed, and subjected to GS-MS using an Agilent Model 7890A/5975C GC–MS system with an Agilent DB-WAX capillary GC column (30 m × 0.25 mm × 0.25 µm). The fatty acid profile comprised distinct molecular species existing in both free and esterified forms.

### Statistical analysis

Statistical analysis was carried out with IBM SPSS version 27 (IBM Corp., Armonk, NY, USA). All statistical comparisons were performed using 25 °C as the control group, and pairwise comparisons were conducted between the control group and two temperature treatment groups (35 °C and 40 °C). A one‑way ANOVA was applied to the data. Prior to conducting the ANOVA, normality was evaluated using Q‑Q plots and Shapiro‑Wilk tests, and homogeneity of variance was verified via Levene’s test. Non-normal data were log-transformed, and Welch’s ANOVA was employed if heteroscedasticity was detected. For datasets meeting the assumptions of normality and homoscedasticity, Fisher’s least significant difference (LSD) test was chosen for post-hoc comparisons, given the controlled experimental conditions with balanced sample sizes, to sensitively detect differences between treatment means while controlling Type I error. Statistical significance was set at *p* < 0.05. Heatmaps with dendrograms were generated using Origin (version 2024; OriginLab, Northampton, MA, USA). To better visualize the variation trends of each lipid or fatty acid species across different treatment groups, Origin automatically performed row-wise Z-score normalization on the mean values of biological replicates during the heatmap plotting process. Accordingly, the values presented on the color scale are these normalized results.

## Results

### High-temperature treatments significantly reduced chlorophyll content

Under heat conditions, *C. reticulata* exhibited significant declines in chlorophyll a, chlorophyll b, and total chlorophyll (a + b) content compared to the control (Table 1).

**Table 1:** Contents of chlorophylls and carotenoids in *Camellia reticulata* under three temperature conditions: 25 °C (Control), 35 °C (H35), and 40 °C (H40)

Parameters		Treatment	
	Control	H35	H40
Chlorophyll a	1.498 ± 0.088 a	1.224 ± 0.191 b	0.933 ± 0.210 c
Chlorophyll b	0.593 ± 0.045 a	0.473 ± 0.065 b	0.391 ± 0.044 c
Chlorophyll (a + b)	2.090 ± 0.130 a	1.697 ± 0.255 b	1.324 ± 0.249 c
Chlorophyll a/b	2.531 ± 0.09 a	2.586 ± 0.097 a	2.365 ± 0.353 a

Values are presented as mean ± SD (*n* = 5). Contents of chlorophyll a, chlorophyll b, total chlorophyll (a + b), and carotenoid are expressed as mg g^−1^ fresh weight (FW). The chlorophyll a/b ratio is dimensionless. A one-way analysis of variance was conducted. Different lowercase letters within the same row indicate significant differences among treatments based on Fisher’s least significant difference at *p* < 0.05. Values shown are the mean ± SD, *n* = 5

*FW* Fresh weight

The H35 treatment significantly reduced these pigments by 18.2%, 20.2%, and 18.8%, respectively, whereas H40 caused more severe reductions of 37.7%, 34.0%, and 36.7%. In contrast, the chlorophyll a/b ratio remained stable across all treatments.

No significant alterations in leaf coloration or morphology were observed in H35-treated plants (Supplementary Fig. S1).

In contrast, H40 treatment triggered visible leaf yellowing that intensified during recovery, and leaf margins gradually curled upward. Despite these symptoms, buds remained undamaged, enabling subsequent new leaf growth.

### High-temperature conditions impaired photochemical activity

During the heat treatments, maximum quantum yield (Fv/Fm) and actual photochemical efficiency [Y(II)] declined progressively with rising temperature, reaching minimal values at 40 °C (Table 2).

**Table 2:** Chlorophyll fluorescence parameters in *Camellia reticulata* under three temperature conditions: 25 °C (Control), 35 °C (H35), and 40 °C (H40)

Parameters		Treatment	
	Control	H35	H40
Fv/Fm	0.775 ± 0.011 a	0.741 ± 0.013 b	0.634 ± 0.039 c
Y(II)	0.334 ± 0.036 a	0.220 ± 0.041 b	0.116 ± 0.025 c
Y(NPQ)	0.392 ± 0.053 b	0.448 ± 0.048 a	0.443 ± 0.046 a
Y(NO)	0.274 ± 0.020 c	0.332 ± 0.027 b	0.442 ± 0.057 a

A one-way analysis of variance was conducted. Different letters indicate significant differences between treatments based on Fisher’s least significant difference at *p* < 0.05. Values shown are the mean ± SD, *n* = 5

*Fv/Fm* Maximum quantum yield, *Y(II)* Actual photochemical efficiency, *Y(NO)* Indicative of non-regulated non-photochemical energy dissipation, *Y(NPQ)* Representing regulated non-photochemical energy dissipation

Specifically, compared with the control, H35 treatment reduced Fv/Fm and Y(II) by 4.4% and 34.1%, respectively. Under H40 treatment, these parameters declined more severely by 18.2% and 65.4%, respectively. Under both H35 and H40 conditions, Y(NPQ) (representing regulated non-photochemical energy dissipation) increased significantly by 14.3% and 12.9%, respectively, while Y(NO) (indicative of non-regulated non-photochemical energy dissipation) increased significantly by 21.1% and 61.2%, respectively.

### The heat treatments resulted in enhanced TAC

**Fig. 1:**
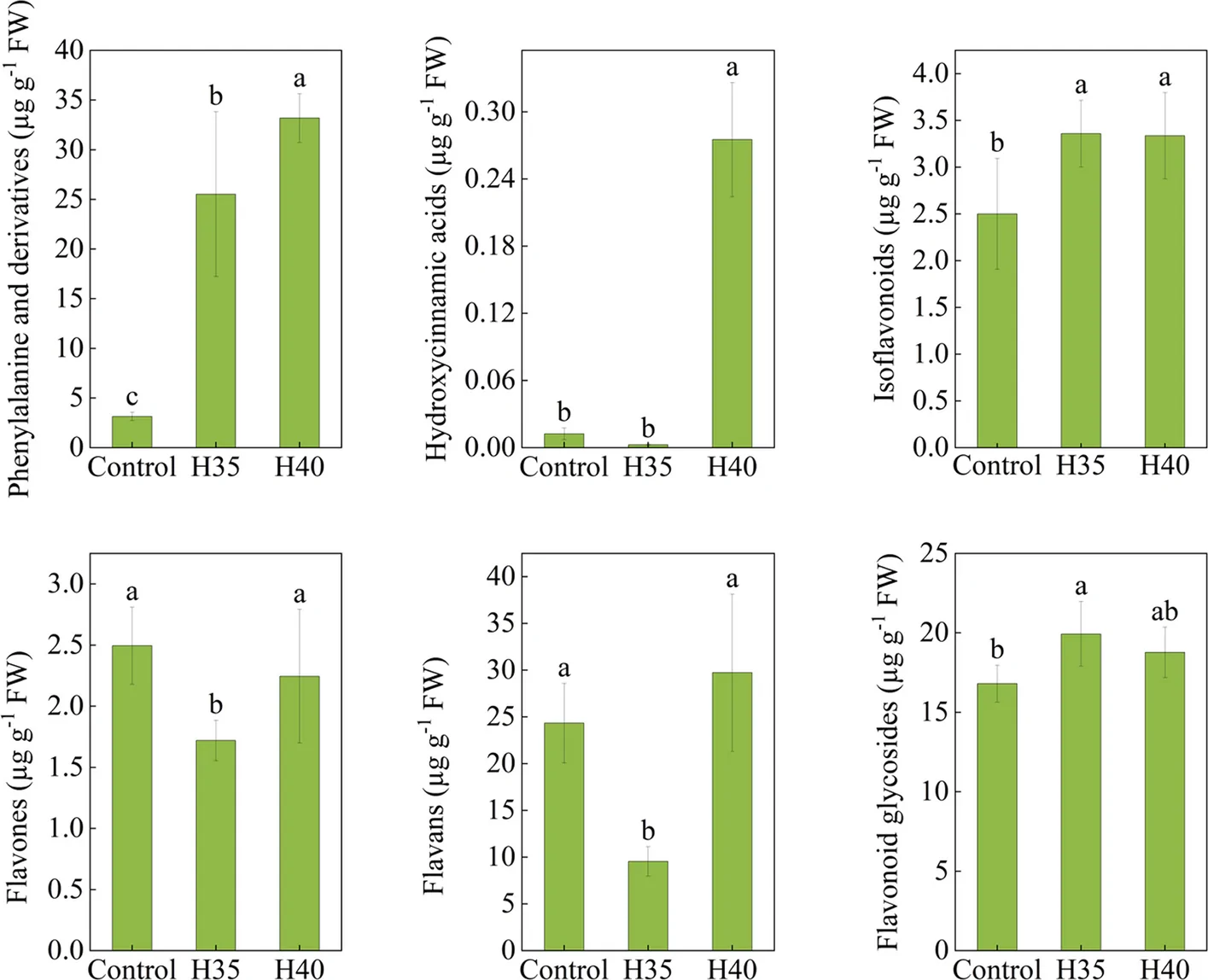
Content of flavonoid biosynthesis pathway-related metabolites under different temperature treatments. Plants of *Camellia reticulata* were subjected to three temperature conditions: 25 °C (Control), 35 °C (H35), and 40 °C (H40). A one-way analysis of variance was conducted. Different letters indicate significant differences between treatments based on Fisher’s least significant difference at *p* < 0.05. Values shown are the mean ± SD, *n* = 5. FW: Fresh weight

Phenylalanine and derivative content significantly increased by 710.5% under H35 and by 954.0% under H40 compared with the control (Fig. 1).

**Fig. 2:**
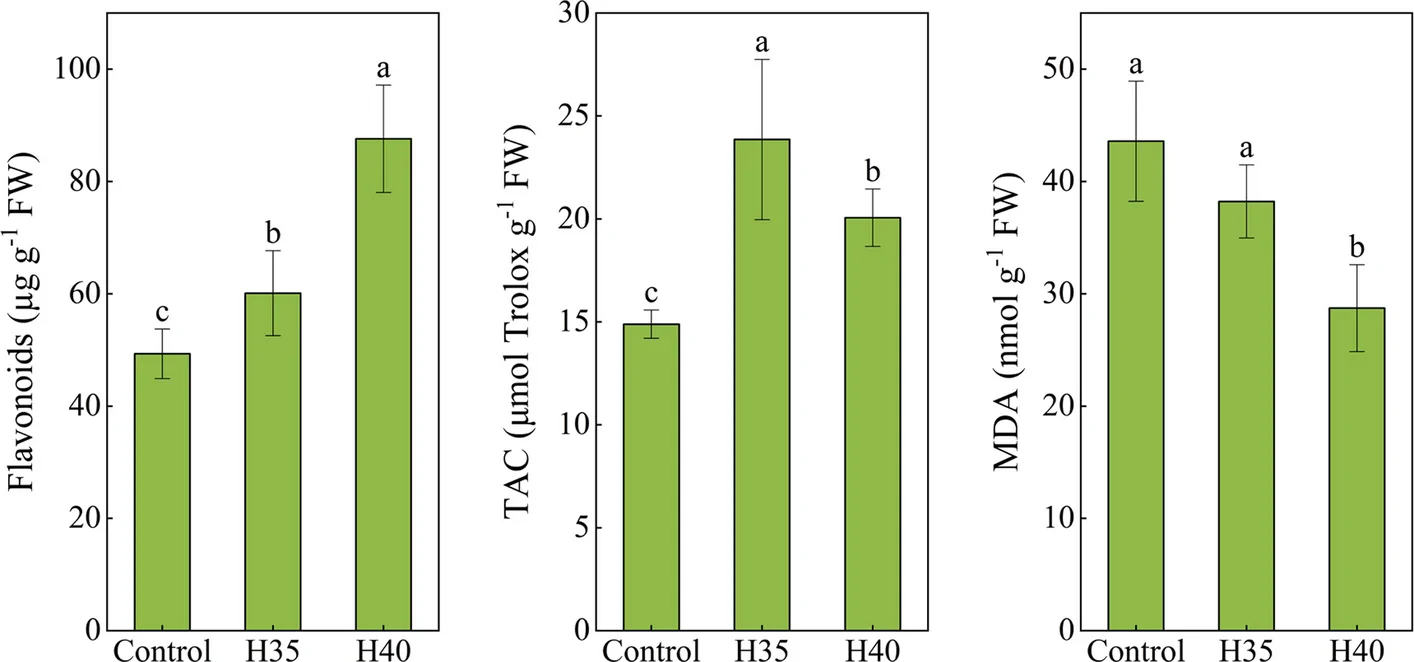
Contents of total flavonoids, total antioxidant capacity (TAC), and malondialdehyde (MDA) under different temperature treatments. Plants of *Camellia reticulata* were subjected to three temperature conditions: 25 °C (Control), 35 °C (H35), and 40 °C (H40). A one-way analysis of variance was conducted. Different letters indicate significant differences between treatments based on Fisher’s least significant difference at *p* < 0.05. Values shown are the mean ± SD, *n* = 5. FW: Fresh weight

Hydroxycinnamic acids content showed no change H35 conditions but significantly increased by 2136.8% under H40 conditions. Isoflavonoids increased significantly under both H35 (34.3%) and H40 (33.4%) conditions. Both flavones and flavans exhibited no significant alteration at H40 while decreasing markedly at H35 stress, by 31.1% and 60.7%, respectively. Flavonoid glycosides increased significantly by 18.6% under H35, while remaining unchanged under H40. Total flavonoid content increased significantly by 21.9% under H35 conditions and by 77.6% under H40 conditions (Fig. 2).

TAC showed marked rises of 60.3% and 34.7% under H35 and H40 treatments, respectively. MDA content remained at control levels under H35 conditions but decreased by 34.1% under H40 conditions.

### Leaf glycerolipid dynamics under heat conditions

**Fig. 3:**
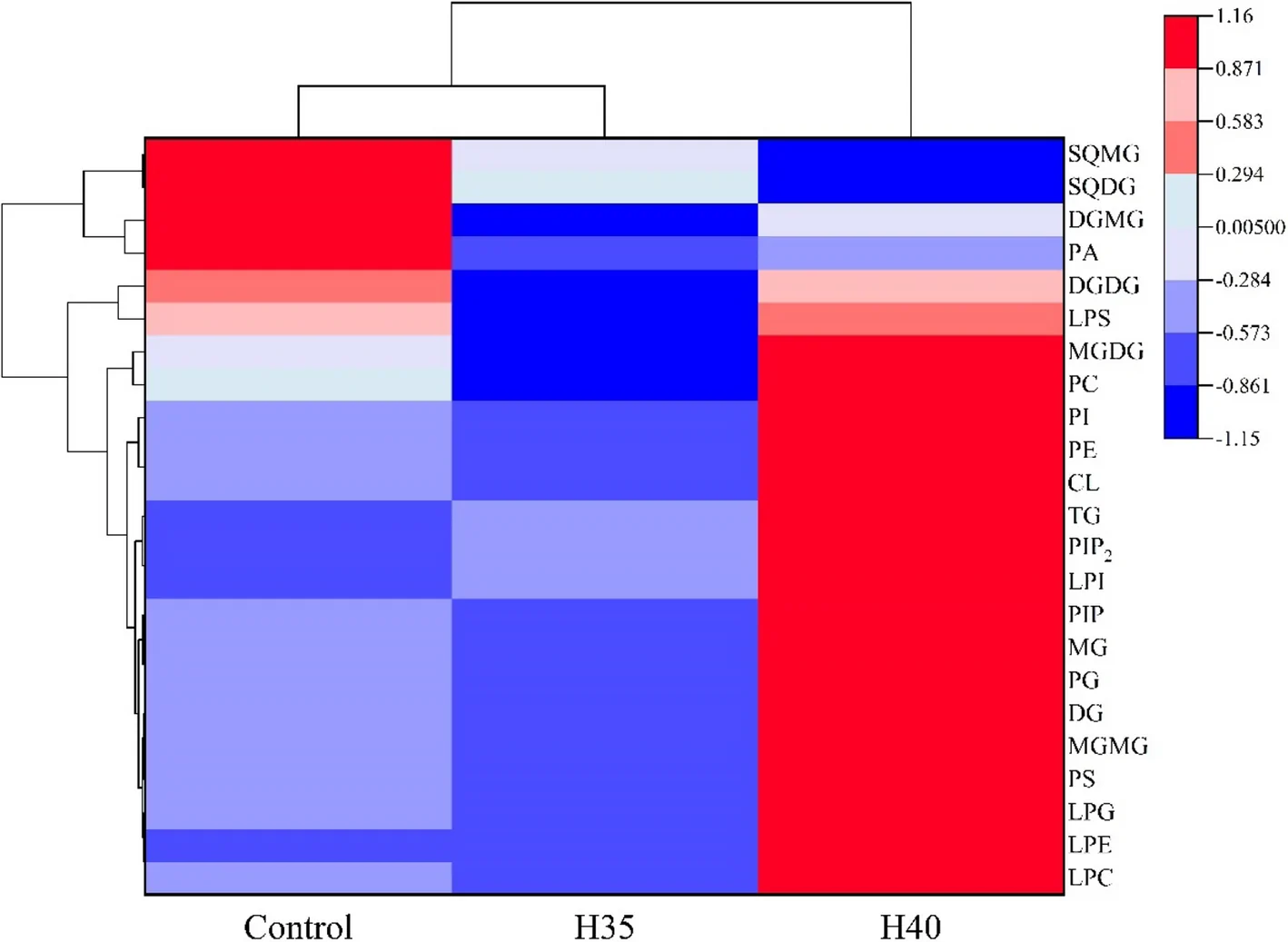
Heatmap of glycerolipid class content under different temperature treatments. Plants of *Camellia reticulata* were subjected to three temperature conditions: 25 °C (Control), 35 °C (H35), and 40 °C (H40). Each column represents a specific treatment, while each row corresponds to an individual lipid class. Color intensity indicates content levels, with red representing higher levels and blue representing lower levels. Data are presented as the means of five biological replicates. Row‑wise Z‑score normalization was automatically performed by Origin on the mean values during heatmap plotting. The color scale represents Z‑score normalized values, ranging from − 1.15 to 1.16, with red indicating higher relative abundance and blue indicating lower relative abundance

Most glycerolipid classes showed significant accumulation under H40 stress (Fig. 3).

**Fig. 4:**
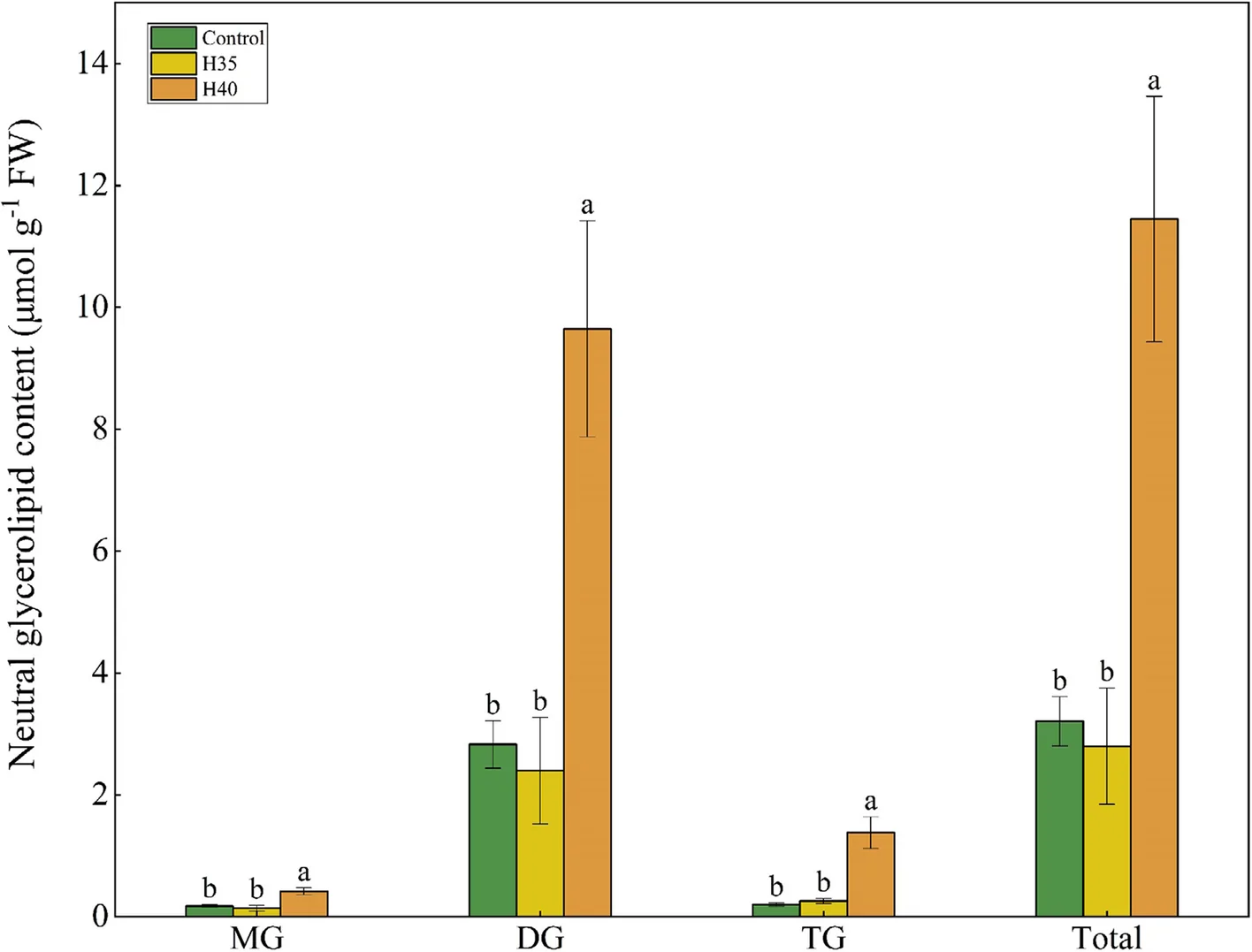
Content of neutral glycerolipid classes under different temperature treatments. Plants of *Camellia reticulata* were subjected to three temperature conditions: 25 °C (Control), 35 °C (H35), and 40 °C (H40). A one-way analysis of variance was conducted. Different letters indicate significant differences between treatments based on Fisher’s least significant difference at *p* < 0.05. Values shown are the mean ± SD, *n* = 5. FW: Fresh weight; MG: Monoacylglycerols; DG: Diacylglycerol; TG: Triacylglycerol

While monoglycerides (MG), diglycerides (DG), and triglycerides (TG) remained stable under H35 conditions compared to the control, their levels increased significantly under H40 stress, by 136.0%, 241.2%, and 582.6%, respectively (Fig. 4).

Total neutral glycerolipid levels significantly increased by 256.9% after exposure to H40 stress. Furthermore, high-temperature exposure significantly elevated the TG/DG ratio, by 56.7% under H35 and 97.6% under H40 (Supplementary Fig. S2).

**Fig. 5:**
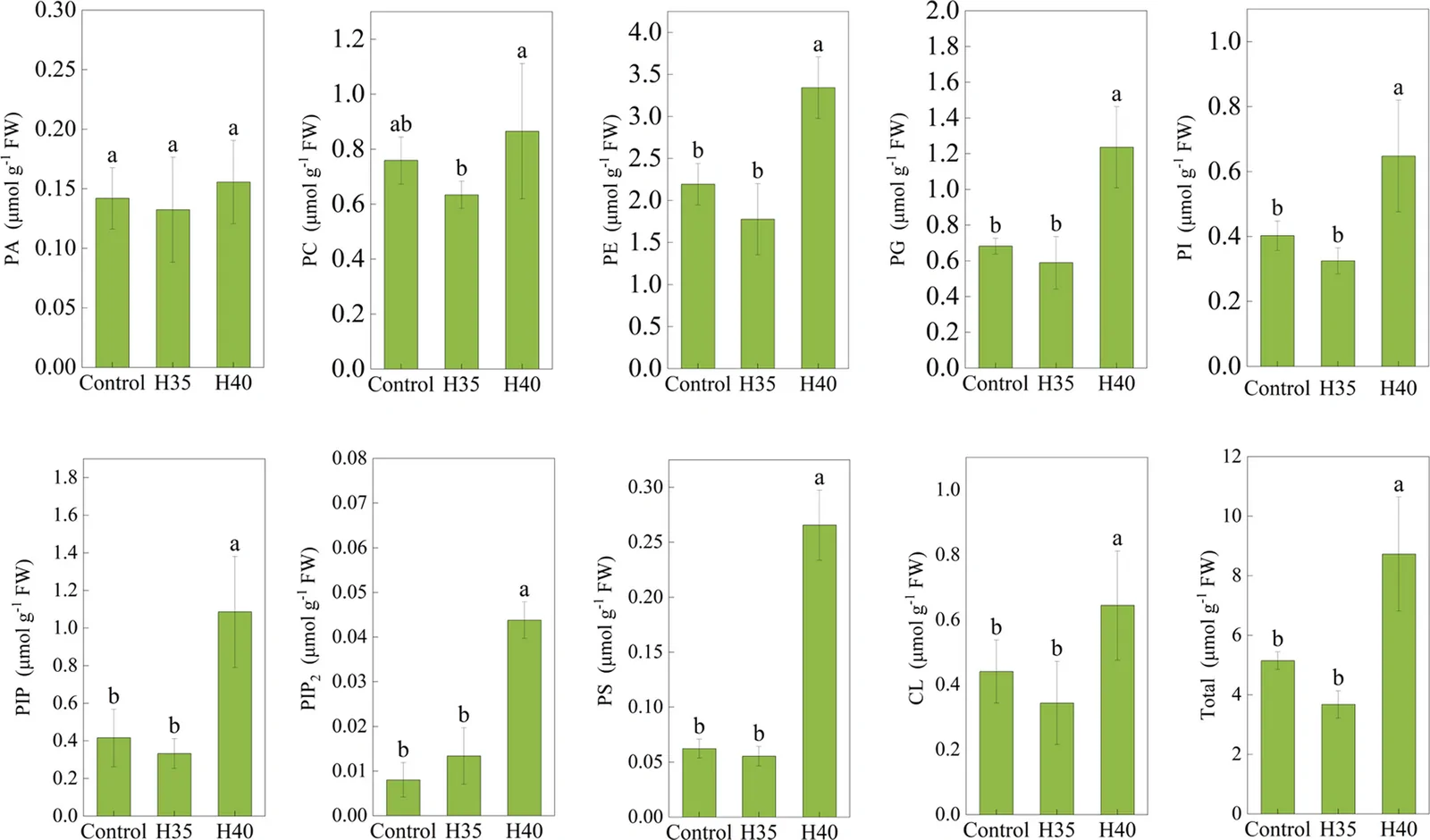
Content of phospholipid classes under different temperature treatments. Plants of *Camellia reticulata* were subjected to three temperature conditions: 25 °C (Control), 35 °C (H35), and 40 °C (H40). A one-way analysis of variance was conducted. Different letters indicate significant differences between treatments based on Fisher’s least significant difference at *p* < 0.05. Values shown are the mean ± SD, *n* = 5. FW: Fresh weight; PA: Phosphatidic acid; PC: Phosphatidylcholine; PE: Phosphatidylethanolamine; PG: Phosphatidylglycerol; PS: Phosphatidylserine; PI: Phosphatidylinositol; PIP: Phosphatidylinositol phosphate; PIP_2_: Phosphatidylinositol 4,5-bisphosphate; CL: Cardiolipin

Phosphatidic acid (PA) and phosphatidylcholine (PC) levels showed no significant differences from the control following high-temperature treatment (Fig. 5).

While unaltered by H35 treatment, all other phospholipid classes accumulated significantly under H40 conditions. Total phospholipid content was unchanged under H35 but significantly increased by 69.5% under H40. The ratio of PC to phosphatidylethanolamine (PE) was unaffected by H35 treatment but decreased significantly following H40 exposure (Supplementary Fig. S2). Lysophosphatidylserine (LPS) content decreased significantly under H35 conditions and showed no change under H40 conditions (Table 3).

**Table 3:** Contents of lysophospholipid species in Camellia reticulata under three temperature conditions: 25 °C (Control), 35 °C (H35), and 40 °C (H40)

Parameters		Treatment	
	Control	H35	H40
LPC	0.00943 ± 0.00180 b	0.00902 ± 0.00180 b	0.06278 ± 0.02579 a
LPE	0.00248 ± 0.00082 b	0.00238 ± 0.00041 b	0.05112 ± 0.02228 a
LPG	0.01487 ± 0.00211 b	0.01365 ± 0.00182 b	0.07294 ± 0.03590 a
LPI	0.00822 ± 0.00351 b	0.01173 ± 0.00480 b	0.04689 ± 0.02237 a
LPS	0.00006 ± 0.00001 a	0.000011 ± 0.000005b	0.00006 ± 0.00003 a
Total	0.03505 ± 0.00478 b	0.03691 ± 0.00524 b	0.23379 ± 0.06826 a

A one-way analysis of variance was conducted. Different letters indicate significant differences between treatments based on Fisher’s least significant difference at *p* < 0.05. Values shown are the mean ± SD, *n* = 5. Due to the low abundance of certain lipid species, values are presented with five decimal places for better clarity

*FW* Fresh weight, *LPC* Lysophosphatidylcholine, *LPE* Lysophosphatidylethanolamine, *LPG* Lysophosphatidylglycerol, *LPI* Lysophosphatidylinositol, *LPS* Lysophosphatidylserine

In comparison, lysophosphatidylcholine (LPC), lysophosphatidylethanolamine (LPE), lysophosphatidylglycerol (LPG), and lysophosphatidylinositol (LPI) did not change after H35 treatment but accumulated significantly after H40 treatment, with increases of 565.5%, 1963.5%, 390.5%, and 470.3%, respectively. Consequently, total lysophospholipid content was unchanged under H35 conditions but significantly increased by 567.1% under H40 conditions.

**Fig. 6:**
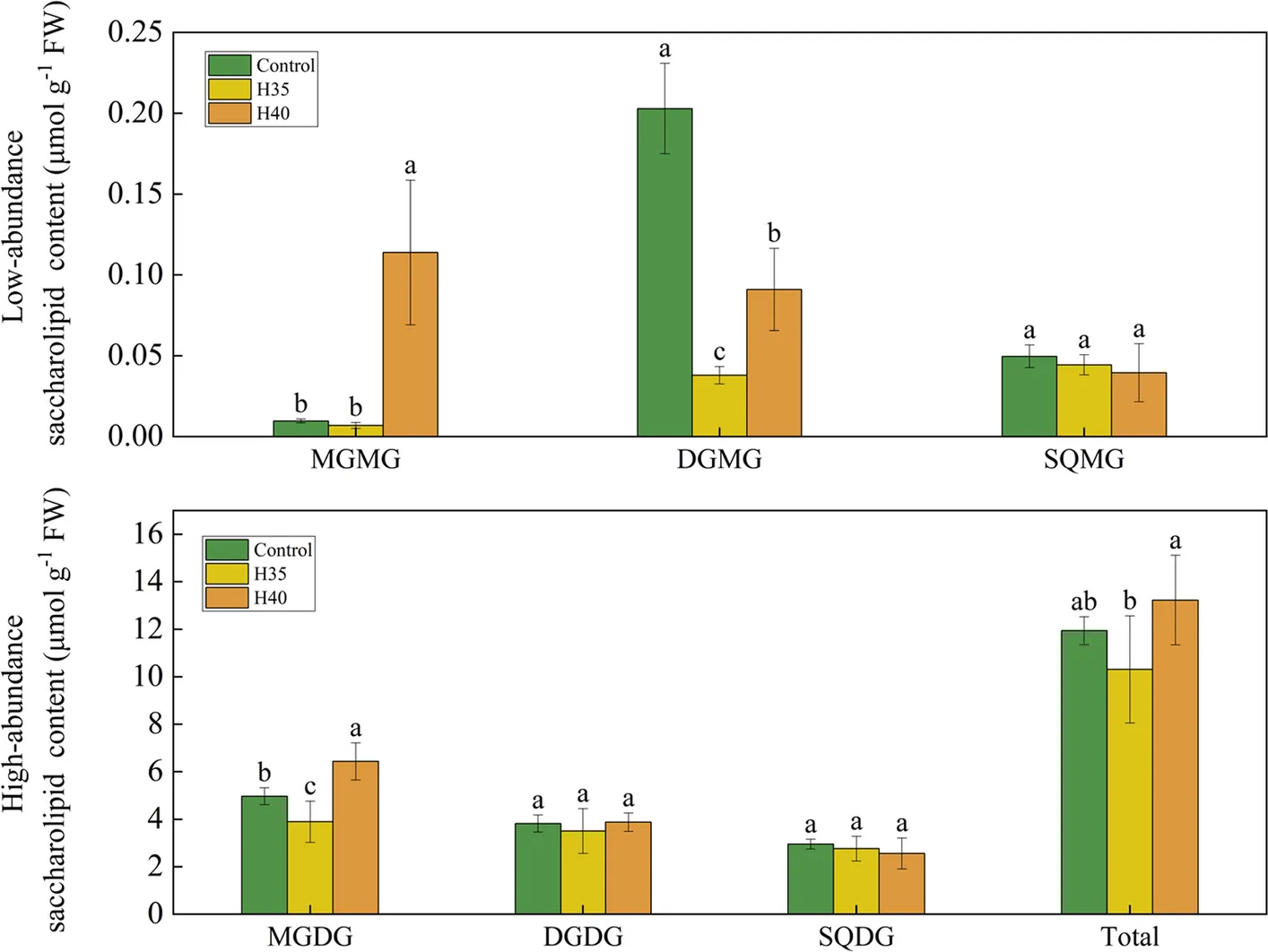
Content of saccharolipid classes under different temperature treatments. Plants of *Camellia reticulata* were subjected to three temperature conditions: 25 °C (Control), 35 °C (H35), and 40 °C (H40). A one-way analysis of variance was conducted. Different letters indicate significant differences between treatments based on Fisher’s least significant difference at *p* < 0.05. Values shown are the mean ± SD, n = 5. FW: Fresh weight; MGMG: Monogalactosylmonoacylglycerol; MGDG: Monogalactosyldiacylglycerol; DGMG: Digalactosylmonoacylglycerol; DGDG: Digalactosyldiacylglycerol; SQMG: Sulfoquinovosylmonoacylglycerol; SQDG: Sulphoquinovosyldiacylglycerol

The contents of digalactosyldiacylglycerol (DGDG), sulfoquinovosylmonoacylglycerol (SQMG), and sulfoquinovosyldiacylglycerol (SQDG) remained unchanged across all treatments (Fig. 6).

In contrast, compared with the control, monogalactosylmonoacylglycerol (MGMG) and monogalactosyldiacylglycerol (MGDG) increased significantly under H40 conditions (by 1069.8% and 29.6%, respectively), whereas under H35 conditions, MGMG was unaffected and MGDG significantly decreased by 21.6%. Digalactosylmonoacylglycerol (DGMG) content declined significantly, by 81.3% under H35 and 55.2% under H40. The DGDG/MGDG ratio significantly increased by 16.0% under H35 but significantly decreased by 21.4% under H40 (Supplementary Fig. S2).

### Degree of unsaturation in individual glycerolipid classes under heat conditions

**Fig. 7:**
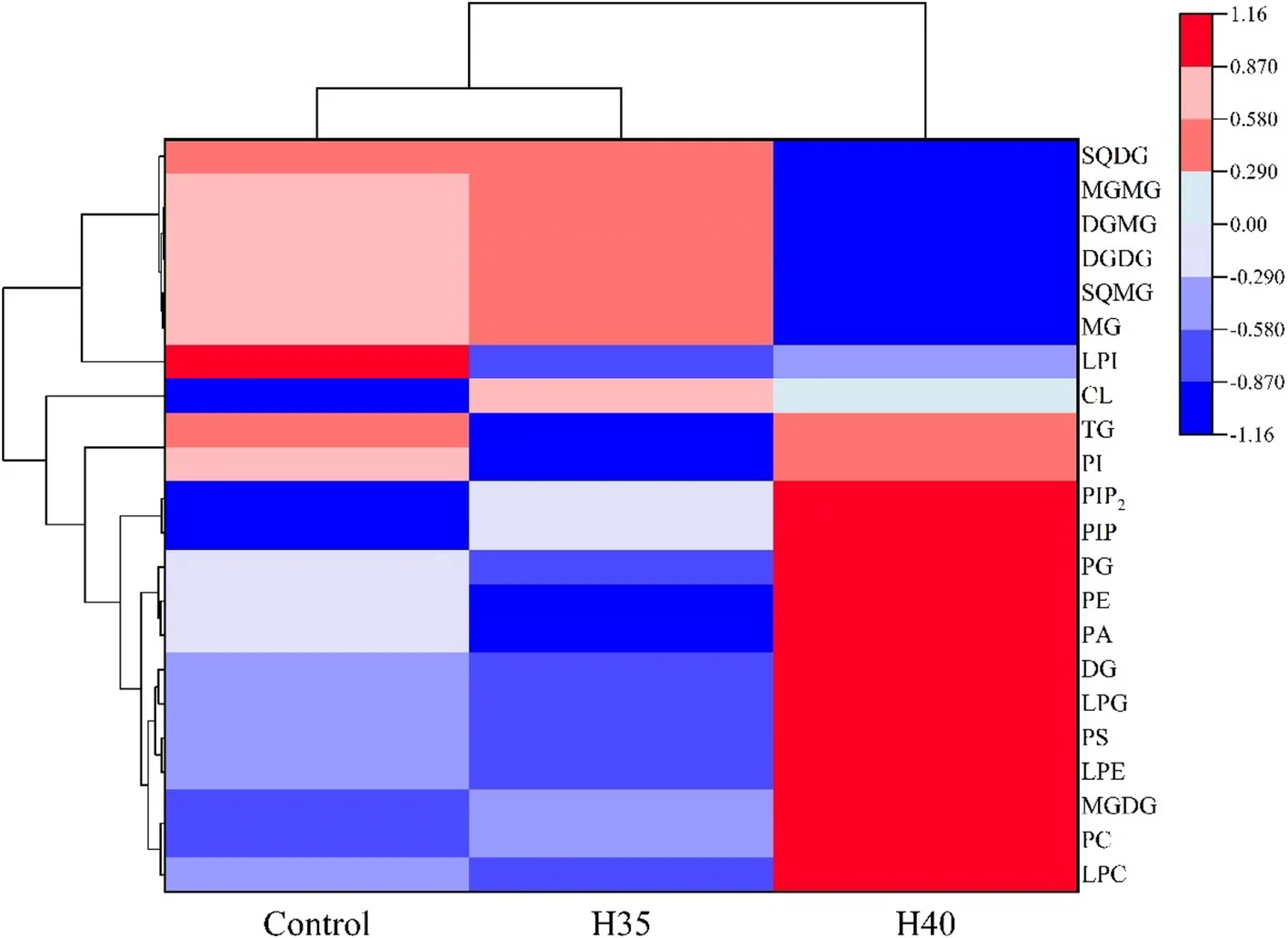
Heatmap of the double bond index (DBI) for glycerolipid classes under different temperature treatments. Plants of *Camellia reticulata* were subjected to three temperature conditions: 25 °C (Control), 35 °C (H35), and 40 °C (H40). Each column represents a specific treatment, while each row corresponds to an individual lipid class. Color intensity indicates DBI levels, with red representing higher levels and blue representing lower levels. Data are presented as the means of five biological replicates. Row‑wise Z‑score normalization was automatically performed by Origin on the mean values during heatmap plotting. The color scale represents Z‑score normalized values, ranging from − 1.16 to 1.16, with red indicating higher relative abundance and blue indicating lower relative abundance

The most significant difference in DBI was observed between the H40 treatment and the control group (Fig. 7).

Relative to controls, diacylglycerol (DG) DBI increased significantly following H40 treatment, whereas monoacylglycerol (MG) and triacylglycerol (TG) DBIs were unaffected by any treatment (Table 4). The total neutral glycerolipid DBI did not change significantly under H35 treatment but increased significantly under H40 treatment.

**Table 4:** Double bond index (DBI) of lipids in *Camellia reticulata* under three temperature conditions: 25 °C (Control), 35 °C (H35), and 40 °C (H40)

Parameters		Treatment	
	Control	H35	H40
Neutral glycerolipids			
MG	2.341 ± 0.059 a	2.340 ± 0.042 a	2.309 ± 0.015 a
DG	2.775 ± 0.037 b	2.657 ± 0.293 b	3.418 ± 0.112 a
TG	6.108 ± 0.212 a	6.019 ± 0.276 a	6.108 ± 0.091 a
Total	2.963 ± 0.053 b	2.968 ± 0.224 b	3.704 ± 0.104 a
Phospholipids			
PA	2.705 ± 0.578 a	2.559 ± 0.405 a	2.926 ± 0.198 a
PC	2.293 ± 0.089 b	2.329 ± 0.114 b	3.055 ± 0.265 a
PE	3.688 ± 0.204 b	3.525 ± 0.233 b	3.982 ± 0.135 a
PG	2.384 ± 0.060 b	2.336 ± 0.085 b	2.489 ± 0.068 a
PI	3.562 ± 0.054 a	2.800 ± 0.622 b	3.559 ± 0.058 a
PIP	1.155 ± 0.351 c	1.680 ± 0.174 b	2.712 ± 0.065 a
PIP_2_	1.914 ± 0.822 b	2.521 ± 0.496 b	3.554 ± 0.169 a
PS	4.460 ± 0.174 b	4.313 ± 0.108 b	5.019 ± 0.416 a
CL	10.472 ± 0.208 a	10.577 ± 0.355 a	10.546 ± 0.190 a
Total	3.630 ± 0.300 b	3.198 ± 0.290 c	3.979 ± 0.037 a
Lysophospholipids			
LPC	0.283 ± 0.084 b	0.276 ± 0.062 b	1.218 ± 0.212 a
LPE	0.723 ± 0.351 b	0.535 ± 0.044 b	1.307 ± 0.065 a
LPG	0.147 ± 0.043 b	0.118 ± 0.036 b	0.472 ± 0.081 a
LPI	1.348 ± 0.217 a	1.249 ± 0.345 a	1.250 ± 0.338 a
Total	0.503 ± 0.118 b	0.516 ± 0.059 b	0.996 ± 0.071 a
Saccharolipids			
DGDG	5.976 ± 0.129 a	5.921 ± 0.297 a	5.293 ± 0.583 b
DGMG	6.281 ± 1.286 a	5.990 ± 1.274 a	3.296 ± 1.707 b
MGMG	3.039 ± 0.138 a	3.012 ± 0.105 a	2.743 ± 0.073 b
MGDG	4.856 ± 0.153 b	4.900 ± 0.181 b	5.726 ± 0.169 a
SQMG	3.539 ± 0.103 a	3.454 ± 0.041 a	2.168 ± 0.178 b
SQDG	4.307 ± 0.315 a	4.306 ± 0.250 a	3.490 ± 0.420 b
Total	5.099 ± 0.118 a	5.094 ± 0.214 a	5.116 ± 0.271 a

Plants of *Camellia reticulata* were subjected to three temperature conditions: 25 °C (Control), 35 °C (H35), and 40 °C (H40). A one-way analysis of variance was conducted. Different letters indicate significant differences between treatments based on Fisher’s least significant difference at *p* < 0.05. Values shown are the mean ± SD, *n* = 5

*MG* Monoacylglycerols, *DG* Diacylglycerol, *TG* Triacylglycerol, *PA* Phosphatidic acid, *PC* Phosphatidylcholine, *PE* Phosphatidylethanolamine, *PG* Phosphatidylglycerol, *PS* Phosphatidylserine, *PI* Phosphatidylinositol, *PIP* Phosphatidylinositol phosphate, *PIP*_*2*_ Phosphatidylinositol 4,5-bisphosphate, *CL* Cardiolipin, *LPC* Lysophosphatidylcholine, *LPE* Lysophosphatidylethanolamine, *LPG* Lysophosphatidylglycerol, *LPI* Lysophosphatidylinositol, *LPS* Lysophosphatidylserine, *MGMG* Monogalactosylmonoacylglycerol, *MGDG* Monogalactosyldiacylglycerol, *DGMG* Digalactosylmonoacylglycerol, *DGDG* Digalactosyldiacylglycerol, *SQMG* Sulfoquinovosylmonoacylglycerol, *SQDG* Sulphoquinovosyldiacylglycerol

Compared with the control plants, PA and cardiolipin (CL) DBIs showed no significant changes under high-temperature conditions (Table 4). The DBIs of PC, PE, phosphatidylglycerol (PG), phosphatidylinositol bisphosphate (PIP_2_), and phosphatidylserine (PS) were unaffected by H35 treatment but significantly increased following H40 exposure. Phosphatidylinositol (PI) DBI decreased significantly following H35 treatment but showed no significant change under H40 conditions. Phosphatidylinositol monophosphate (PIP) DBI increased significantly under both heat conditions. The DBI of total phospholipid declined significantly under H35 but increased significantly under H40.

Relative to the controls, LPC, LPE, and LPG showed no significant changes under H35 treatment but significantly increased under H40 treatment (Table 4). LPI DBI remained unaltered across all treatments. Total lysophospholipid DBI remained stable under H35 but increased significantly under H40.

The DBIs of DGDG, DGMG, MGMG, SQDG, and SQMG remained stable under H35 compared to controls but decreased significantly under H40 (Table 4). In contrast, the DBI of MGDG significantly increased after H40 treatment. The total saccharolipid DBI remained unchanged across all high-temperature treatments.

### Fatty acid composition changed significantly under extreme high temperature

**Fig. 8:**
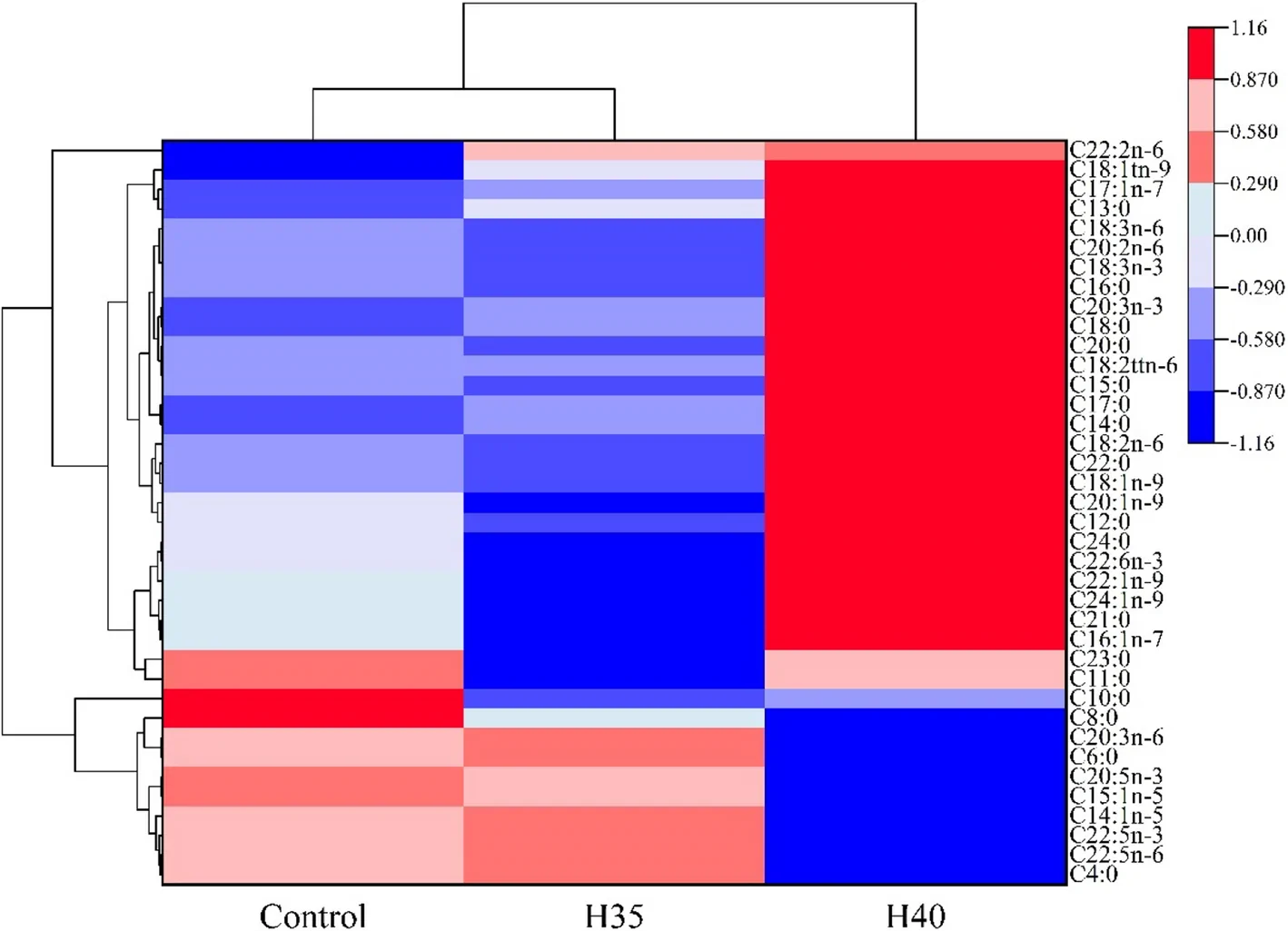
Heatmap of individual fatty acid species content under different temperature treatments. Plants of *Camellia reticulata* were subjected to three temperature conditions: 25 °C (Control), 35 °C (H35), and 40 °C (H40). Each column represents a specific treatment, while each row corresponds to an individual fatty acid species. Color intensity indicates content levels, with red representing higher levels and blue representing lower levels. Data are presented as the means of five biological replicates. Fatty acid nomenclature: number of carbon atoms followed by number of double bonds (position of the first double bond from the methyl end, n-x). E.g., C20:3 n-6 denotes a 20-carbon fatty acid with 3 double bonds, with the first double bond located at the sixth carbon from the methyl end. Row‑wise Z‑score normalization was automatically performed by Origin on the mean values during heatmap plotting. The color scale represents Z‑score normalized values, ranging from − 1.16 to 1.16, with red indicating higher relative abundance and blue indicating lower relative abundance

Fatty acid species showed the most pronounced changes under H40 conditions (Fig. 8).

*Trans*-fatty acids (C18:1tn-9, C18:2ttn-6) and odd-numbered fatty acids were detected. The content of C18:1tn-9 increased significantly relative to the control under both H35 and H40 conditions, by 67.3% and 174.7%, respectively (Supplementary Fig. S3).

**Fig. 9:**
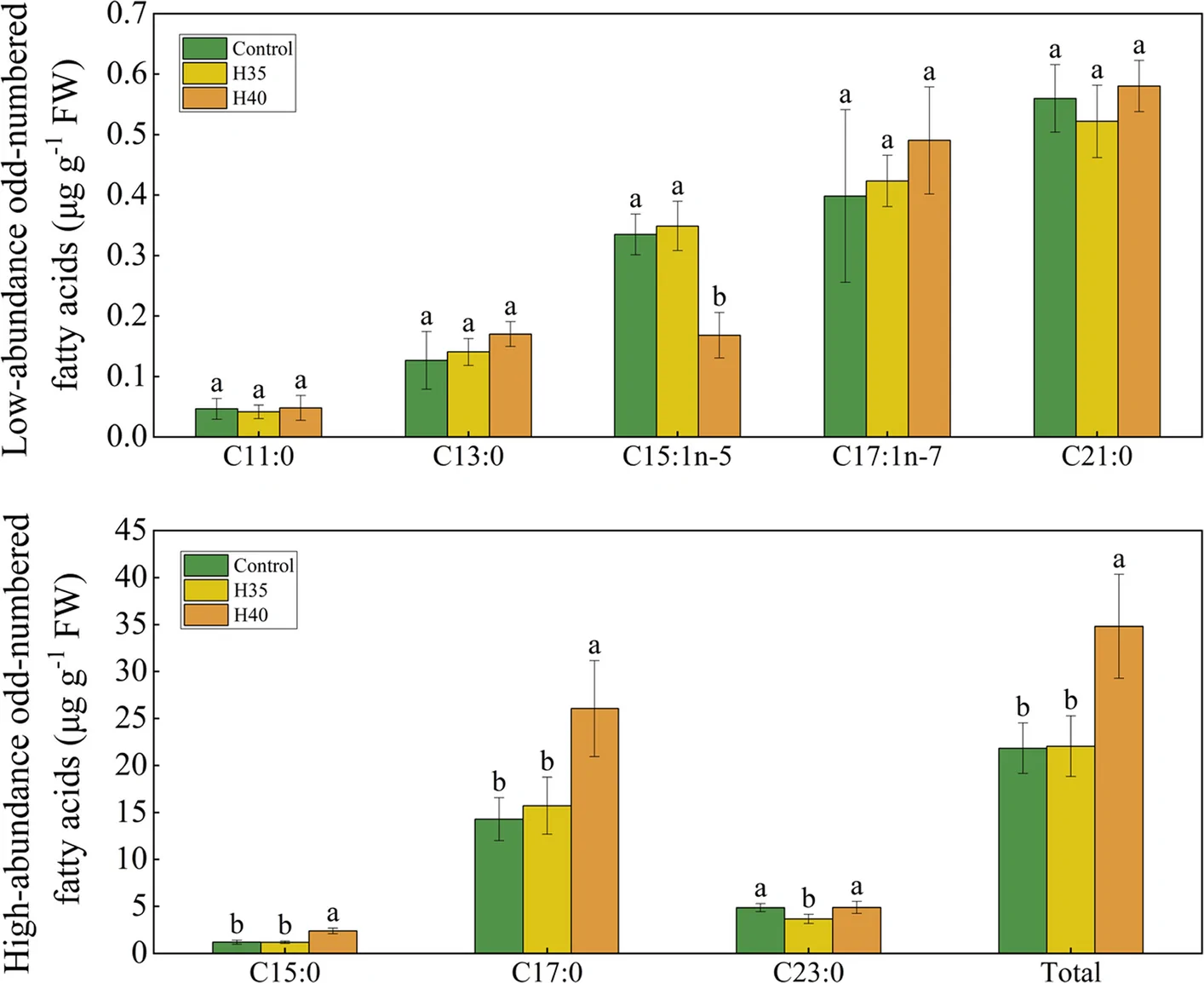
Content of odd-numbered fatty acid species under different temperature treatments. Plants of *Camellia reticulata* were subjected to three temperature conditions: 25 °C (Control), 35 °C (H35), and 40 °C (H40). A one-way analysis of variance was conducted. Different letters indicate significant differences between treatments based on Fisher’s least significant difference at *p* < 0.05. Values shown are the mean ± SD, *n* = 5. Fatty acid nomenclature: number of carbon atoms followed by number of double bonds (position of the first double bond from the methyl end, n-x). E.g., C15:1n-5 denotes a 15-carbon fatty acid with 1 double bond, with the first double bond located at the fifth carbon from the methyl end. FW: Fresh weight

In contrast, C18:2ttn-6 content showed no significant change under H35 but significantly increased by 66.7% under H40. Odd-numbered fatty acids were unaffected by H35 treatment but significantly accumulated by 59.4% under H40 conditions (Fig. 9).

**Fig. 10:**
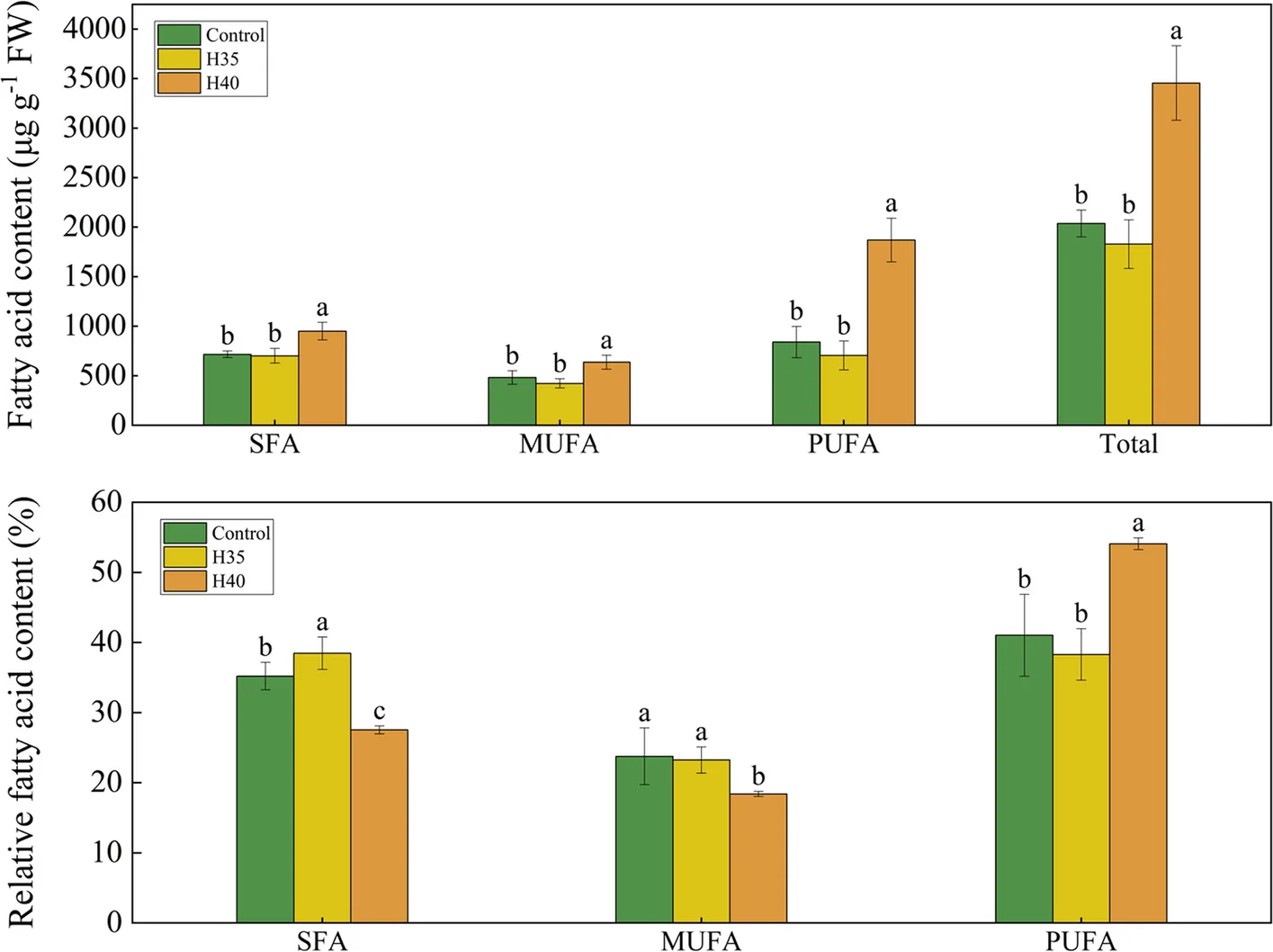
Content and relative content of fatty acids grouped by unsaturation level under different temperature treatments. Plants of *Camellia reticulata* were subjected to three temperature conditions: 25 °C (Control), 35 °C (H35), and 40 °C (H40). A one-way analysis of variance was conducted. Different letters indicate significant differences between treatments based on Fisher’s least significant difference at *p* < 0.05. Values shown are the mean ± SD, *n* = 5. FW: Fresh weight; SFA: Saturated fatty acids; MUFA: Monounsaturated fatty acids; PUFA: Polyunsaturated fatty acids

The levels of SFAs, MUFAs, and PUFAs (saturated, monounsaturated, and polyunsaturated fatty acids, respectively) showed no significant changes compared with the control under H35 treatment. However, H40 treatment significantly elevated the contents of SFA, MUFA, and PUFA by 32.7%, 31.9%, and 122.6%, respectively (Fig. 10).

The content of total fatty acids was unaffected under H35 but increased significantly by 69.6% under H40. Under H35 conditions, the relative content of SFA increased significantly, while those of MUFA and PUFA remained unaltered (Fig. 10).

Conversely, under H40 conditions, the relative content of PUFA increased significantly, with concurrent decreases in those of SFA and MUFA.

## Discussion

High-temperature stress triggers multifaceted antioxidant responses to alleviate oxidative injury in plants [23, 38]. MDA is widely recognized as a marker of oxidative lipid injury [20], and its significant accumulation under heat stress has been demonstrated as a biochemical indicator of aggravated oxidative damage [39]. However, its role is complex, as some studies report a lack of correlation between MDA content and stress intensity [40, 41], and others suggest it may also serve a protective function [21, 42]. In the present study on *C. reticulata*, MDA levels remained unchanged under H35 treatment but decreased significantly under H40 treatment, accompanied by a significant increase in TAC. Although such a response is not commonly reported, the non-elevation or even reduction in MDA under high temperature has been documented across various plant species. In the congeneric species *C. nitidissima*, MDA content exhibited no significant increase following 24 h of heat stress at 45 °C [43]. Furthermore, in *Helianthus annuus*, MDA levels in roots and chloroplasts remained unaltered or even declined after 1 h of heat shock at 40 °C [44]. Flavonoids exhibit a remarkable diversity of biochemical properties crucial for plant survival and adaptation, as they both inhibit the generation of and quench existing reactive oxygen species [45].

By this mechanism, enhanced biosynthesis of flavonoids can effectively alleviate oxidative stress-induced damage, a defense response that may be partially reflected by reduced MDA accumulation [46, 47]. Therefore, heat-induced flavonoid accumulation may have partially contributed to the observed increase in TAC in *C. reticulata*. Taken together, the decrease in MDA under extreme heat in *C. reticulata* is not an anomaly, but rather the result of multiple synergistic protective mechanisms: substantial flavonoid accumulation together with enhanced TAC effectively suppresses ROS-driven peroxidation initiation. The enzymatic detoxification of MDA by aldo–keto reductases (AKRs) and aldehyde dehydrogenases (ALDHs) reported in the literature also provides a broader biochemical context for the dynamic changes in MDA levels [48, 49]. The regulation of this antioxidant system likely lays the foundation for post-stress recovery—under H40 treatment, despite leaf browning, the buds remained viable and capable of sprouting; whereas under H35 treatment, no visible morphological changes were observed. The fact that leaf browning was not prevented, yet bud viability was maintained, indicates that the decrease in MDA is not equivalent to an overall alleviation of oxidative damage, but rather more likely reflects the metabolic signature of a coordinated biochemical survival strategy adopted under extreme heat. This strategy reflects a prioritization of stress adaptation over normal developmental programs, a phenomenon also observed in reproductive tissues under heat stress [50].

Chlorophylls are essential for photosynthesis, initiating energy transduction via light absorption and excited electron generation [51]. Given this central role, chlorophyll degradation constitutes the most prominent biochemical change during both natural and stress-induced leaf senescence [52]. In the present study, high-temperature stress markedly reduced chlorophyll content in *C. reticulata*, suggesting accelerated degradation and/or inhibited biosynthesis under heat conditions. Notably, regulated chlorophyll catabolism has been suggested to alleviate phototoxic damage under stress by reducing light absorption and avoiding excessive energy excitation, thereby limiting ROS formation [53]. Although pronounced chlorophyll degradation occurred under both H35 and H40 treatments, accompanied by partial leaf browning at H40, all plants remained viable. This survival, together with the coordinated reduction in MDA and elevation in TAC, implies that heat-induced chlorophyll decline may serve as an adaptive strategy to minimize photo-oxidative risk. However, under H40, the more pronounced leaf browning observed after the recovery period suggests that chlorophyll loss may have transitioned from a regulated photoprotective response to irreversible photodamage.

Photosystem II (PSII), an essential element in plant photosynthetic process, exhibits strong sensitivity to environmental stress [54]. When light absorption exceeds the capacity for photochemical utilization, excess excitation energy can lead to photoinhibitory processes [7]. In this study, both H35 and H40 treatments caused significant reductions in Fv/Fm and Y(II). These declines are consistent with the occurrence of photoinhibition. However, we note that under H40, sustained photoprotective quenching could also contribute to the observed decreases [55]. Notably, leaf browning was absent under H35 but visible under H40, paralleling the greater loss of photochemical efficiency. Both heat treatments decreased Y(II) while simultaneously increasing Y(NPQ) and Y(NO), suggesting that excess excitation energy was partitioned into both regulated and non-regulated dissipation pathways. Under H35, the significant increase in Y(NPQ) was accompanied by a rise in Y(NO), indicating that the upregulation of photoprotective capacity was quantitatively insufficient to fully dissipate the excess energy. The absence of leaf browning under this condition further suggests that the photoprotective apparatus remained largely operational. In contrast, under H40, Y(NPQ) was not significantly different from H35, yet Y(NO) increased much more drastically. This shift may reflect a limitation in the photoprotective machinery that goes beyond a mere quantitative insufficiency. Several, non-mutually exclusive factors could account for this pattern, such as a reduced transthylakoid proton gradient, limited activity of the xanthophyll cycle, heat-induced alterations in thylakoid membrane organization, or changes in feedback regulation of energy dissipation [49]. Thus, the increased proportion of Y(NO) suggests impaired energy dissipation processes. However, it remains speculative to attribute this response specifically to damage of the xanthophyll cycle or thylakoid membrane structure based on our current data. The fact that browning became even more pronounced after the recovery period under H40 further indicates that extreme heat caused irreparable damage to the photosynthetic apparatus. Future work will need to directly assess chloroplast ultrastructure and the integrity of photosystem components to verify the exact targets of severe heat stress.

Our lipidomic data further reveal that *C. reticulata* employs distinct membrane remodeling and resource management strategies under different heat intensities, representing a lipid-centered shift from adaptation to survival that parallels the physiological transition from "coordinated acclimation" to "dysregulated state" observed at the whole-plant level. Lipids play pivotal roles in plant growth and development, as well as environmental stress responses through regulation of membrane composition, energy metabolism, and signal transduction [17, 25, 26, 32].

Many plant species adapt to temperature fluctuations by adjusting the unsaturation level of membrane lipids. To mitigate temperature-induced changes in membrane fluidity, organisms generally upregulate acyl desaturation under low temperatures and downregulate it under high temperatures [26]. MGDG and PE are cone-shaped lipids that tend to form non-bilayer structures, whereas DGDG and PC adopt a cylindrical conformation to stabilize bilayer structures [18, 29]. Furthermore, TG contributes to membrane stabilization and photoinhibition alleviation, while DG facilitates the formation of non-bilayer structures [17, 56]. Accordingly, elevated DGDG/MGDG, PC/PE, and TG/DG ratios collectively stabilize the membrane bilayer, thereby preserving membrane integrity under heat stress [29]. Under H35, the increase in DGDG/MGDG ratio, the elevated TG/DG ratio, and decreased phospholipid unsaturation indicate a conservative, adaptive response that enhances membrane stability and aligns with established thermotolerance mechanisms [56, 57]. This response differs fundamentally from that under H40, which triggered extensive metabolic reprogramming toward lipid metabolism. Under H40, we observed the co-accumulation of neutral glycerolipids, phospholipids, and lysophospholipids. Neutral glycerolipids, particularly TG, which are essential for energy storage and fatty acid metabolic homeostasis, accumulated significantly [58–60], likely serving as a protective sink for excess fatty acids released from membrane remodeling. The increase in phospholipid content may represent a compensatory response aimed at enhancing membrane biosynthetic capacity to support the maintenance of extraplastidic membrane integrity under heat stress. Consistent with this notion, a similar accumulation of phospholipids contributing to membrane stabilization has been reported in some heat-tolerant species [61, 62]. The significant accumulation of lysophospholipids under H40 indicates their involvement in signal cascades triggered by severe heat stress [63], while also reflecting membrane damage caused by phospholipid hydrolysis. Targeted fatty acid profiling revealed that this was underpinned by a significant increase in total fatty acid content and a marked shift in composition, notably a substantial rise in PUFAs. The coordinated accumulation of diverse lipid classes implies a substantial rerouting of carbon toward lipid metabolism, representing a prominent metabolic reprogramming strategy adopted by some plants under abiotic stress [30, 64]. Critically, the significant increase in the unsaturation of DG, the key metabolic intermediate positioned at the junction of glycerolipid pathways [65], suggests that the accumulated PUFAs are actively channeled through this central biosynthetic hub. We propose that channeling PUFAs through the DG pool serves a dual purpose: sequestration into the expanding TG pool (reflected in the markedly enhanced TG/DG ratio) to mitigate peroxidation risk, and direct incorporation into membranes for essential restructuring. This restructuring is characterized by a suite of changes that collectively reduce membrane stability. It is noteworthy that while increased lipid unsaturation is adaptive under cold stress [18, 26], excessive unsaturation under heat stress can conversely destabilize membranes by disrupting the lipid-protein interactions and supramolecular organization vital for the function and stability of photosynthetic complexes [18, 66]. Such changes are evident in extraplastidic membranes as a decreased PC/PE ratio and increased phospholipid unsaturation, and in plastidic membranes as accumulation of unsaturated MGDG and a decreased DGDG/MGDG ratio. The shift in PC/PE and DGDG/MGDG ratios increases the proportion of non-bilayer-prone lipids (PE and MGDG), whereas the concurrent increase in lipid unsaturation enhances membrane fluidity [18, 29]. Together, these changes create a dysregulated lipid environment under H40. Specifically, the marked increase in phospholipid unsaturation was accompanied by a decrease in the unsaturation of specific saccharolipid classes (DGDG, DGMG, MGMG, SQMG, SQDG), indicating a compartment-specific, targeted adjustment of membrane properties. This suggests that extraplastidic and plastidic membranes undergo distinct remodeling patterns under extreme heat. Given the well-documented asymmetric distribution of lipids in membrane microdomains [27, 67], these specific decreases in the unsaturation of some saccharolipid classes may help maintain local membrane stability by compensating for the increased unsaturation in MGDG.

Furthermore, the appearance of unusual lipids, including trans-unsaturated and odd-numbered fatty acids, reveals a novel aspect of thermoadaptation. The remarkable accumulation of trans isomers (C18:1tn-9, C18:2ttn-6) under H40 suggests a potential mechanism for membrane adaptation. Given that cis-to-trans isomerization is known to reduce membrane fluidity in bacteria exposed to organic toxins [68, 69], a similar process in *C. reticulata* may represent a conserved strategy to modulate membrane physical state under abiotic stress. In parallel, we observed a significant H40-induced biosynthesis of odd-numbered fatty acids. The detected species included C11:0, C13:0, C15:0, C15:1n-5, C17:0, C17:1n-7, C21:0, and C23:0. Under H40 stress, the total pool of odd-numbered fatty acids increased markedly. This finding extends recent observations of their accumulation under heat stress in other plant species [70], reinforcing the potential link between odd-numbered fatty acid metabolism and thermotolerance in plants. While the precise roles of both trans isomers and odd-numbered fatty acids in plant membranes require validation, their co-accumulation indicates that *C. reticulata* activates unique metabolic pathways to adjust membrane properties under extreme heat conditions.

## Conclusion

*C. reticulata* employs distinct adaptive strategies in response to varying heat intensities. Under H35, the plant avoided severe oxidative damage and visible tissue injury by enhancing its antioxidant capacity to maintain oxidative homeostasis and by remodeling lipids to enhance membrane stability, as evidenced by an increased DGDG/MGDG ratio, decreased phospholipid unsaturation, and an elevated TG/DG ratio. In contrast, H40 treatment triggered a survival-oriented resource reallocation, where the plant prioritized massive synthesis of flavonoids and sequestration of lipids into neutral glycerolipids. This strategy successfully suppressed lipid peroxidation (as shown by reduced MDA) and ensured bud survival, despite severe PSII photodamage, visible leaf discoloration, and increased phospholipid unsaturation. These findings illuminate the adaptive mechanisms that enable *C. reticulata* to withstand high temperatures and provide a physiological framework for future investigations into the molecular regulation of these pathways. From a practical perspective, it is recommended that regions with summer maximum temperatures below 35 °C be prioritized for the introduction and cultivation of *C. reticulata*, although field validation experiments are still required. A limitation of this study is that lipidomic and physiological measurements were collected only at a single time point (after 3 days of heat stress). Future time-course sampling across both stress and recovery phases would help distinguish adaptive lipid remodeling from heat-induced damage and clarify the linkage between metabolic dynamics and the development of visible injury.

## Supplementary Information

Supplementary Material 1.

## Data Availability

Data will be made available on request.

## Abbreviations

ROS: Reactive Oxygen Species
PSII: Photosystem II
MDA: Malondialdehyde
TAC: Total Antioxidant Capacity
DBI: Double bond index
FW: Fresh weight
Fv/Fm: Maximum quantum yield
Y(II): Actual photochemical efficiency
Y(NPQ): Representing regulated non-photochemical energy dissipation
Y(NO): Indicative of non-regulated non-photochemical energy dissipation
MG: Monoglyceride
DG: Diglyceride
TG: Triglyceride
PA: Phosphatidic acid
PC: Phosphatidylcholine
PE: Phosphatidylethanolamine
PG: Phosphatidylglycerol
PI: Phosphatidylinositol
PIP: Phosphatidylinositol(4)phosphate
PIP2: Phosphatidylinositol(4,5)bisphosphate
PS: Phosphatidylserine
CL: Cardiolipin
LPC: Lysophosphatidylcholine
LPE: Lysophosphatidylethanolamine
LPG: Lysophosphatidylglycerol
LPI: Lysophosphatidylinositol
LPS: Lysophosphatidylserine
DGDG: Digalactosyldiacylglycerol
DGMG: Digalactosylmonoacylglycerol
MGMG: Monogalactosylmonoacylglycerol
MGDG: Monogalactosyldiacylglycerol
SQMG: Sulfoquinovosylmonoacylglycerol
SQDG: Sulfoquinovosyldiacylglycerol
SFA: Saturated Fatty Acid
MUFA: Monounsaturated Fatty Acid
PUFA: Polyunsaturated Fatty Acid
