# Supplementary material for: Multi-level responses of Camellia reticulata to heat stress: insights from photosynthetic performance, antioxidant activity, lipidomics, and fatty acid profiling
Source: BMC Plant Biol. 2026 Jun 15;26:1435. doi: 10.1186/s12870-026-09072-x (PMC13495249; doi:10.1186/s12870-026-09072-x)
Supplement: Supplementary file 1 [file 12870_2026_9072_MOESM1_ESM.docx]

**Fig. S1.** Leaf morphology of *Camellia reticulata* immediately after temperature treatments (25°C, 35°C, 40°C) and after a 10-day recovery period. Panels a, b, c display leaf morphology immediately after heat stress at 25°C, 35°C, 40°C, respectively; panels d, e, f show leaf morphology after a 10-day recovery following heat stress at 25°C, 35°C, 40°C, respectively.

**Fig. S2.** The ratios of different lipid classes in *Camellia reticulata* under three temperature conditions: 25°C (Control), 35°C (H35), and 40°C (H40). A one-way analysis of variance was conducted. Different letters indicate significant differences between treatments based on Fisher’s least significant difference at *p* ˂ 0.05. Values shown are the mean ± SD, n = 5. DG: Diacylglycerol; DGDG: Digalactosyldiacylglycerol; MGDG: Monogalactosyldiacylglycerol; PC: Phosphatidylcholine; PE: Phosphatidylethanolamine; TG: Triacylglycerol.

Fig. S3. Content of trans-fatty acid species under different temperature treatments.

Plants of *Camellia reticulata* were subjected to three temperature conditions: 25°C (Control), 35°C (H35), and 40°C (H40). A one-way analysis of variance was conducted. Different letters indicate significant differences between treatments based on Fisher’s least significant difference at *p* ˂ 0.05. Values shown are the mean ± SD, n = 5. FW: Fresh weight.
